# Protocol of a clinical trial study involving educational intervention in patients treated with warfarin

**DOI:** 10.1097/MD.0000000000015829

**Published:** 2019-05-31

**Authors:** Josiane Moreira da Costa, Milena Soriano Marcolino, Heloisa Carvalho Torres, Raissa Eda de Resende, Renan Pedra de Souza, Hannah Cardoso Barbosa, Daniel Dias Ribeiro, Maria Auxiliadora Parreiras Martins

**Affiliations:** a Faculdade de Farmácia; b Faculdade de Medicina; c Hospital das Clínicas; d Escola de Enfermagem; e Instituto de Ciências Biológicas, Universidade Federal de Minas Gerais, Belo Horizonte, Minas Gerais, Brazil.

**Keywords:** anticoagulants, clinical trial protocol, developing countries, health education, warfarin

## Abstract

**Background:**

Atrial fibrillation (AF) is the most common sustained arrhythmia worldwide. Oral anticoagulation is an effective strategy for primary and secondary prevention of stroke in patients with AF. Warfarin is an oral anticoagulant widely prescribed and, despite its benefits, the achievement of the goals of drug therapy depends on patient involvement, among other factors. Educational interventions can contribute for effectiveness and safety of oral anticoagulation therapy. We sought to describe the protocol of a clinical trial designed to evaluate the effect of a patient-centered educational strategy focused on low-income patients with AF and poor anticoagulation control.

**Methods:**

Patients ≥18 years with AF, on warfarin for at least 6 months and time in therapeutic range (TTR) <60% will be recruited at 2 anticoagulation clinics (ACs) in Brazil. Patients from 1 AC will be allocated to the intervention group and patients from the other AC will be allocated to the control group. Intervention group will attend educational sessions based on a patient-centered care approach, and the control group will receive usual care. The intervention will be based on Paulo Freire's theory and tailored according to practices involving health empowerment and techniques applied to individuals with limited socioeconomic status. The intervention is estimated to last 5 months. We will consider TTR as the primary outcome and knowledge and self-reported non-adherence to warfarin therapy as secondary outcomes. TTR values and non-adherence will be measured before intervention (T0) and at times immediately after (T1), and 3 (T2), 6 (T3), 9 (T4), and 12 (T5) months after intervention. Knowledge will be measured at times T0, T1 e T5. The calculated sample size indicated 85 patients in each group.

**Discussion:**

The proposed study aims to investigate whether an innovative educational approach to deliver care to a low-income population on warfarin improves anticoagulation control. Once our hypothesis is confirmed, our findings are expected to help improving anticoagulation control, knowledge on warfarin therapy and adherence to drug therapy. Thus, we believe our results may contribute to improve oral anticoagulation effectiveness in a low-income population.

Trial registration: Registro Brasileiro de Ensaios Clínicos (ReBEC) RBR- 9cy6py and UTN: U1111-1217-0151 (March, 2019).

## 1 Introduction

Atrial fibrillation (AF) is the most common sustained arrhythmia worldwide.^[1]^ It is considered a public health challenge, with high comorbidity, increased mortality risk and high incremental health cost.^[1,2]^ It is as an independent factor for stroke,^[1]^ and oral anticoagulation is an effective strategy on primary and secondary prevention of cardioembolic stroke on patients with AF.^[3,4]^ Despite the availability of the direct oral anticoagulants, warfarin is still widely used in this context.^[5]^

Warfarin exhibits a narrow therapeutic index, a wide variability of dose-response and the potential to interact with many drugs and foods containing vitamin K. Adjustments on warfarin dosages are guided by regular monitoring of prothrombin activity, expressed by the International Normalized Ratio (INR). Oral anticoagulation control is challenging due to several aspects, such as INR oscillations, the need of frequent dose adjustments, influence of genetic polymorphisms, non-adherence to drug therapy, among others.^[4]^ Poor anticoagulation control increases the risk of hemorrhagic and cardioembolic events.^[6,7]^

Anticoagulation quality is often measured by the time in therapeutic range (TTR) which is a method useful to identify the proportion of time patients attain INR values within the desired therapeutic target.^[8]^ Low values of TTR are influenced by sociodemographic factors, such as low family income and inadequate health literacy.^[9]^ Average TTR below 60% is associated with higher incidence of hemorrhagic and cardioembolic events.^[6,7]^ Therefore, the assessment of anticoagulation control may help to stratify patients with higher risk of complications. These patients could benefit from patient-centered educational strategies designed to improve their active participation in self-care and drug adherence.

A systematic review performed by Clarkesmith et al^[10]^ pointed out the existence of evidence gaps related to the impact assessment of educational strategies focused on patients taking oral anticoagulants. All studies included in the review were performed in high-income countries and the studies usually exclude patients with inadequate health literacy^[10]^ which would benefit the most from educational interventions.^[9]^ In the context of low- and middle-income countries, some findings have reinforced the need of investments on strategies to improve knowledge on oral anticoagulation.^[11,12]^ In those countries, there are significant barriers that hinder the health education process in vulnerable populations. As an alternative, educational strategies using the principle of empowerment may contribute to encourage patients in self-care and potentially would improve the results of drug therapy. Although there is evidence of impact from patient-centered educational strategies for chronic disease management,^[13,14]^ the evidence is lacking for patients taking warfarin. Thus, we aimed to describe the protocol of a controlled clinical trial designed to evaluate the effect of a patient-centered educational strategy focused on low-income patients with AF and poor anticoagulation control.

## 2 Methods

### 2.1 Study setting

This is a non-randomized controlled clinical trial to be conducted at 2 public university hospitals in Belo Horizonte, a 3.5-million-inhabitant city in Southeast Brazil. Patients will be recruited at 2 anticoagulation clinics (ACs) from the hospitals Hospital das Clínicas da UFMG (HC-UFMG) and Hospital Risoleta Tolentino Neves (HRTN). Patients from the former AC will be allocated to the intervention group and patients from the latter AC will be allocated to the control group.

These study settings are tertiary referral hospitals. More than 90% of patients perform INR at the hospital laboratory. Those ACs are multidisciplinary with a team composed by physicians, pharmacists, and nurses. Specialized care is regularly provided to outpatients who receive education and warfarin dose adjustments. Both ACs have similar anticoagulation protocols to guide professionals to perform standardized care practice. The trial has been registered on Registro Brasileiro de Ensaios Clínicos (REBEC) (http://www.ensaiosclínicos.gov.br). We followed the SPIRIT recommendations to describe the study protocol.^[15]^

### 2.2 Eligibility criteria

Patients assisted at the 2 ACs will be considered as potentially eligible for study participation. The inclusion criteria will be age ≥18 years, both sexes, follow-up in the ACs for at least 6 months, valvular or non-valvular AF as indication for warfarin use and TTR<60%. The exclusion criteria will be bedridden patients or limitation of locomotive disorders that compromises the attendance to office visits; total blindness or deafness; aphasia or other speech disorders that could hinder communication during study; dementia diagnosis reported in medical records; less than 2 INR values in the study period. Relatives or caregivers will be allowed to participate in the intervention, but they will not be assessed in this study. Follow-up loss will be considered as the absence in at least 2 intervention appointments or 2 or more intervention replacements.

### 2.3 Intervention

**Figure 1:**
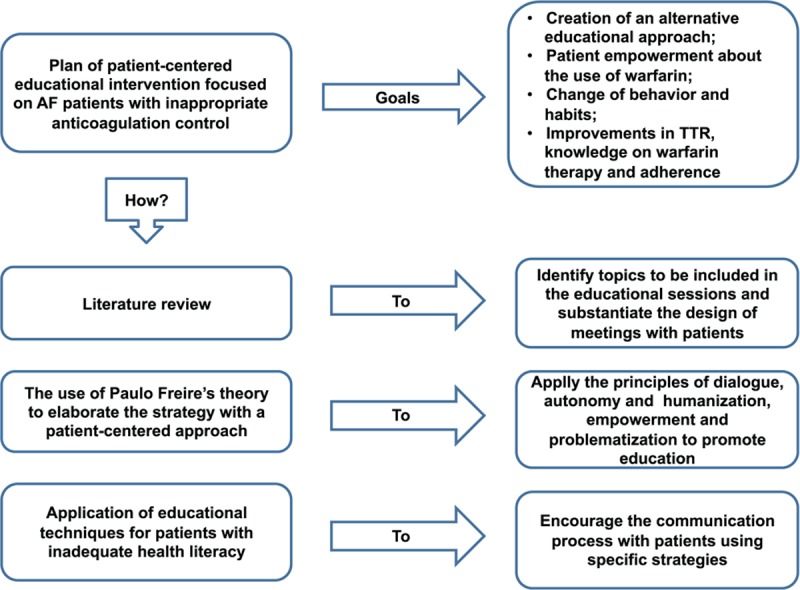
Intervention plan. AF = atrial fibrillation, TTR = time in therapeutic range.

The intervention will be preceded by a broad search in the literature to identify the topics to be included in the educational sessions and to substantiate the design of meetings with patients, as well as the methods indicated to encourage interaction between participants. The literature review included MEDLINE, LILACS, CINAHL, and COCHRANE databases. We considered studies published from 1st July 2007 to 31st January 2018. The intervention strategy was designed to adopt a patient-centered care approach following the Paulo Freire's theory.^[16,17]^ Freire is a Brazilian educator who has developed a theoretical framework based on problematization to promote education. He is widely renowned by his educational concepts that propose the principles of dialogue, autonomy, humanization, and emancipation. We elaborated the intervention using the principle of health empowerment ^[16,18]^ and we will apply techniques adapted to patients with low socioeconomic status and inadequate health literacy ^[19]^ (Fig. 1).

The topics selected for the intervention sessions were: “knowing the medical condition and the anticoagulation indication”, “appropriate use of warfarin and other drugs taken by the patient”, “drug-drug and drug-food interaction?” and “risks and benefits from the treatment”.^[8,20]^

The intervention will be developed in groups of 12 participants at maximum. It will begin with a “culture circle”, defined as a learning method based on “generating words” that reflect the participants’ life experiences. The “generating words” will be identified by an immersion of the researcher in patient care process. Recognition of the context and the patients’ needs about the care process will be carried out. The educator will promote a debate to strongly motivate each group participant. Patients will be encouraged to share their life experiences and fears with other people who may have similar experiences. Thus, they will be able to develop a critical opinion out of their own background.^[16,17]^

Each group of 12 patients will participate in 4 face-to-face meetings, with intervals of approximately 30 days. As the topics will be repeated in each group, patients who miss a meeting will have other opportunities to attend the missing topic. Each meeting will be composed by a warm-up step, followed by the topic presentation and the learning construction. Participants will be invited to exchange experiences about warfarin therapy, to identify their main goals and obstacles, and to set future goals to be pursued. Each meeting is planned to last 1 hour, with a possible extension to 1 hour and a half, when necessary.

One clinical pharmacist trained on health education will conduct the intervention together with supervised Pharmacy undergraduates. Researchers will receive specific training by a senior researcher with experience in the methodology to develop skills in techniques to promote self-care and to stimulate patient empowerment. Pilot educational sessions will be offered to an independent group of patients with similar sociodemographic characteristics and a thrombosis history who are assisted at different week days of the intervention group at HC-UFMG. These patients will not be included in the study analyses. Researchers will not get involved in patient care during the study period, which will be under the responsibility of the AC professionals.

In-between meetings, each patient will receive a telephone call. On the first telephone call, the patients will be asked about their goal related to the treatment and what they have been doing to achieve that. On the second telephone call, they will be asked about warfarin adherence and about personal strategies to not miss warfarin doses. On the third and fourth telephone calls, the patients will be asked about the consumption of foods rich in vitamin K and about the use of other medications taken continuously. In this approach, a protocol will be used to ask questions and information will be registered on a standardized form. Patients will be constantly stimulated to take active participation in the intervention.

**Figure 2:**
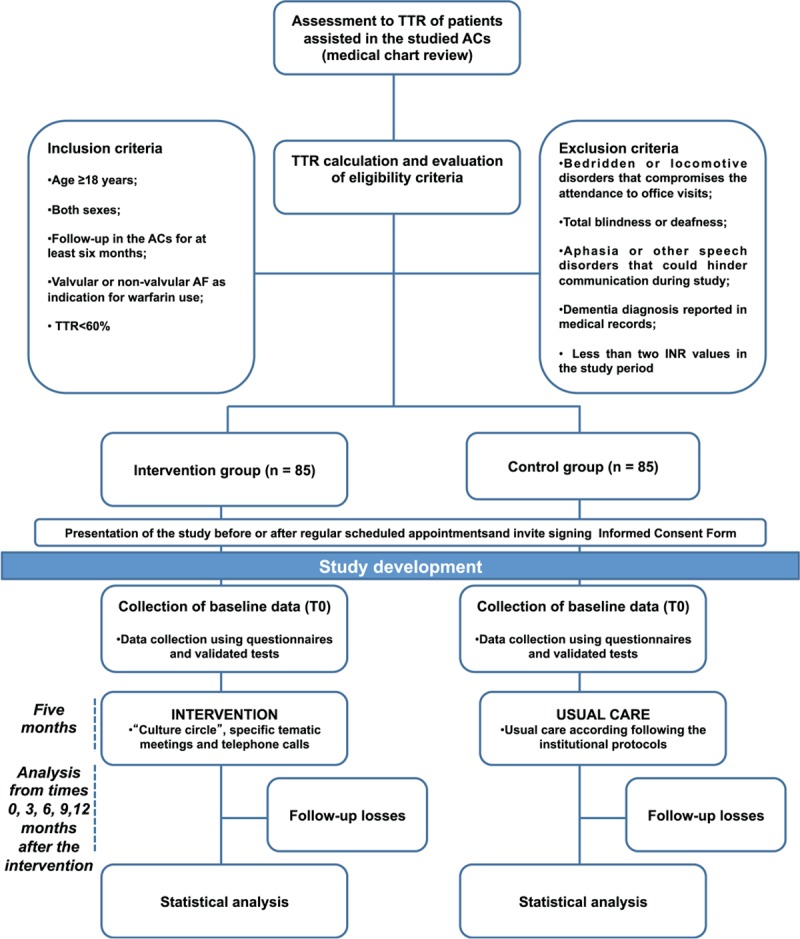
Flow diagram. ACs = anticoagulation clinics, AF = atrial fibrillation, TTR = time in therapeutic range, INR = international normalized ratio.

The pilot study will be conducted in March and April, 2019. The intervention will begin in April and May 2019 and it is planned to be concluded by September and October, 2019. Intervention will last approximately 5 months taking into account extra group sessions to replaced missing intervention meetings. Data collection will conclude by 12 months after the intervention. The study design is depicted in Figure 2.

### 2.4 Outcomes

The primary outcome will be the oral anticoagulation control, assessed by TTR. We will consider as secondary outcomes knowledge on warfarin therapy and self-reported adherence to oral anticoagulation.

**Figure 3:**
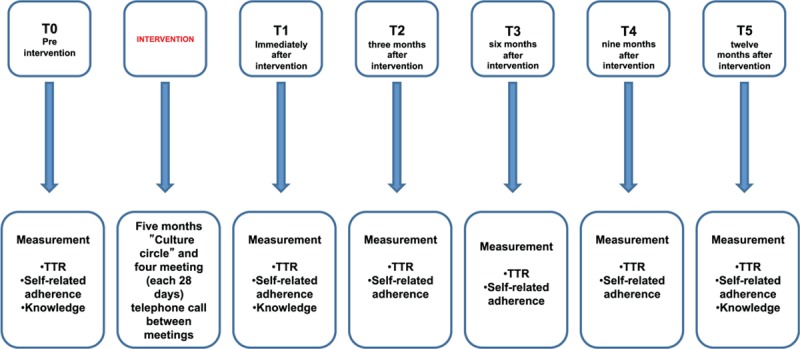
Flowchart of the study phases. T1 = time 1, T2 = time 2, T3 = time 3, T4 = time 4, T5 = time 5, TTR = time in therapeutic range.

The outcomes will be compared intra and intergroups, using pre-defined time points to measure outcomes before and after the intervention. TTR and self-reported adherence to warfarin therapy will be measured 6 months before starting the intervention (T0), immediately after intervention completion (T1) and at 3 (T2), 6 (T3), 9 (T4) and 12 (T5) months after intervention completion. To prevent patients from exhaustion and considering that knowledge takes time to change, this variable will be measured at T0, T1, and T5 (Fig. 3).

We will collect the following data:

(i) sociodemographics: age, sex, family income, cognitive status, dependence on activities of daily living (ADL), religiosity, alcohol consumption, and smoking habits;
(ii) clinical data: indication for warfarin use, INR target, type and number of comorbidities, number of missing appointments, stroke history;
(iii) laboratorial data: INR values; and
(iv) information on drug therapy: number of concomitant drugs used chronically, assistance with taking warfarin or other prescribed medications, warfarin dosage changes, complexity of drug therapy, and modifications in concomitant medications.

### 2.5 Sample size

Sample size was calculated using diggle.linear.power function of longpower package in the software R 3.4.4 version.^[21]^ The TTR will be prospectively assessed, from T0 to T5. We considered an absolute increase of 6% in TTR with standard deviation of 7.5,^[22]^ 80% power, level of significance of 0.05 and at least 15% of variation in the slope coefficient between intervention and control groups. The calculated sample was 71 individuals for each group, and we planned to include 20% additional patients to account for a follow-up loss, resulting in 85 individuals allocated in each group. Also, we used composite symmetry autocorrelation structure with a correlation of 0.80 between the times of data collection.

### 2.6 Recruitment

Before recruitment, all patients assisted between August 2017 and September 2017 at the studied ACs (n = 1371) had their TTR calculated to identify those presenting TTR <60%. A new TTR calculation occurred between July and December 2018 to certify if patients with low TTR (<60%) identified in the first screening would continue to fulfill inclusion criteria. Thus, patients with persistently low TTR will be invited to participate in the study. To complete the sample, new patients with low TTR between July and December 2018 will be considered for inclusion in the study. Recruitment phase started in January and is expected to end by May. Patients’ invitation is planned to take place before or after scheduled appointments for monitoring oral anticoagulation.

### 2.7 Data collection and outcome assessment

Data will be collected by 3 trained pharmaceutical researchers. Data collection in the control group will occur in parallel with the intervention group. The Oral Anticoagulation Knowledge (OAK) Test^[23]^ will be applied to assess knowledge on warfarin therapy. It has been recently validated to be used in the Brazilian population.^[24]^ The instrument consists of 20 questions with 4 possible answers and only 1 correct option. Each correct answer scores 1 point, leading to a total score ranging from 0 to 20 points. Higher scores (>75%) indicate better knowledge levels about warfarin therapy.^[23,24]^

Self-reported adherence will be evaluated by reviewing patient records in the hospital computerized system. It has been deemed to be an adequate method comparable to other instruments applied to assess adherence.^[25,26]^ Non-adherence will be considered as at least 1 missing warfarin doses or administration errors according to prescribed dosing regimen.

Sociodemographic, clinical and drug therapy data will be collected by chart review. Patients will be interviewed to investigate their religiosity, level of dependence on activities of daily living (ADL), cognitive status and health literacy.

Validated instruments translated into Brazilian Portuguese will be used. The Duke Religious Index (DUREL) has 5 items assessing 3 of the religiosity dimensions most related to health outcomes: organizational religiosity (OR), non-organizational religiosity (NOR) and intrinsic religiosity (IR). Scores (OR, NOR, and IR) are evaluated separately and they cannot be used as a total score.^[27]^ Katz scale consists of 6 items that measures the individual performance on self-care activities, according to a complex hierarchy: nourishment, sphincter control, transference, personal hygiene and ability of get dressed and taking baths, to identify dependence level on ADL.^[28]^ Mini-Mental State Examination (MMSE) is divided into 2 sections. The first one requires only vocal answers and includes orientation, memory, and attention with the highest score of 21. The second section tests ability to nominate, to follow verbal and written commands, to write a phrase and to copy a polygon with the highest score of 9.^[29,30]^ Short Assessment of Health Literacy for Portuguese-speaking Adults (SAHLPA-18) contains 18 items allowing the classification of participants as having inadequate or adequate health literacy with scores of 0 to 14 or 15 to 18, respectively. Participants are invited to read out loud 18 medical terms and to associate them to 1 of 2 words present to the participant.^[31]^ At last, the Medication Regimen Complexity Index (MRCI) is a specific, reliable and valid tool used to measure the complexity of pharmacotherapy.^[32]^

### 2.8 Data management

Identification of participants will be coded. The results from the surveys application and from the examination of charts data will be registered in worksheets and will be under the responsibility of the researcher responsible for the research. Database will be validated by double typing using Epi Data, 3.1 software.

### 2.9 Statistical analysis

TTR will be calculated by the Rosendaal method using a linear interpolation of INR results. TTR will be calculated at least 2 INR measurements. For participants who had INR intervals higher than 56 days, we used INR values to calculate TTR for the valid intervals (<56 days) and afterward we used the independent TTR values to calculate the final TTR by the arithmetic mean.^[33]^

Categorical variables will be expressed as frequency and percentage. Symmetric continuous variables will be expressed as means and standard deviation while asymmetric data will be expressed as median, first quartile, and third quartile.

To evaluate significance of each variable in the outcomes, univariate models will be adjusted using generalized estimation equation (GEE) with appropriate distribution for each outcome (normal or gamma distribution for TTR and binomial distribution for knowledge and adherence). Wald tests will be performed for each variable coefficient using an initial significance level of 0.20. All variables identified as initially significant will be added to a multivariate model and significance level will be set at 0.05. One final GEE multivariate model will be presented for each outcome including exclusively significant coefficients. Post hoc analysis will include comparisons of outcome means (or probabilities) at different time points. Statistical analysis will be performed in R version 3.4.4.

Patient refusal, death or other situations that would make it difficult for the patient to continue in the study will be registered and they will be considered as an intention to treat for analysis. Important protocol modifications will be reported to REBEC.

## 3 Discussion

This study will allow assessing the effect of a patient-centered intervention on patients taking warfarin in a population with low socioeconomic status. We will explore critical aspects of warfarin treatment, including TTR, knowledge, and adherence to warfarin therapy. The trial design will help investigating the effect of an educational intervention specifically tailored to empower patients with poor anticoagulation control. We expect to offer an innovative educational approach to help improving patient self-care and, consequently, achieving better therapeutic results.

AF has increased its prevalence worldwide. It is expected that a growing number of patients will have indication for oral anticoagulation, including those with low-income and inadequate health literacy.^[1,34]^ Patient education is a challenge in providing care to warfarin patients with limited socioeconomic status.^[8,9]^ Studies focusing on educational interventions can contribute to the creation of more effective strategies to be applied in patients with inadequate health literacy.

We believe that the employment of the principles of empowerment can help patients making better decisions for self-care. Although widely known, empowerment practices are little explored by studies involving health education in low- and middle-income countries. Paulo Freire's theory has proposed educational strategies that enrich patients’ previous experience, critical thinking, and a dialogical process.^[16,17]^ This must be built by educators and learners. These strategies may contribute to change patients’ behavior toward warfarin therapy and to improve adherence to the treatment and to the recommended diet, and also to increase TTR.

In our study, we will assess a real-world population treated in Brazilian ACs differing from other studies that involved highly selected populations with exclusion of people with inadequate health literacy and cognitive impairment. Previous studies conducted in ACs in Brazil showed that there is a stratum of patients with a persistent TTR <60% who could potentially benefit from health empowerment strategies to improve oral anticoagulation control.^[35,36]^ The strength of our study is that it can fill a gap in the scientific knowledge on the impact of educational interventions focused on low-income patients with AF and poor anticoagulation control. It stands out that low-income populations are not extensively studied. Several studies carried out in developed countries have pointed out difficulties to obtain results with health education actions in patients with inadequate health literacy population on warfarin.^[8,9]^ Another strength is that the settings of our study adopt similar standardized protocols for anticoagulation management bringing consistency to the comparisons of TTR.

To minimize selection bias, we will exclude patients with treatment duration <6 months to improve the homogeneity of the studied groups. We believe that patients using warfarin for <6 months (inception patients) would have different perceptions and behavior towards warfarin therapy than those using for >6 months (experienced patients).^[37]^ The allocation of patients in the intervention or control groups located in separated centers will reduce bias related to the interference of intervention over the control group. Researchers will not get involved in clinical practice during the conduction of the study. Their involvement could interfere in the standardized care provided to the intervention group. The measured outcomes—TTR, knowledge, and adherence to warfarin therapy—will be assessed at different time intervals, allowing short- and long-term evaluations of impact of the educational intervention. It is expected that the final results from this study will be available by July 2020.

As limitations, we should address that the studied patients will be randomized intragroup, but not intergroup. Moreover, we will not identify definitive outcomes, such as hemorrhagic and thromboembolic complications. This derives from the limitation of data collection regarding the history of previous of INR values and occurrence of adverse events. Patients who presented adverse events may have been admitted by other healthcare institutions hindering the access to patient clinical history.

Our study will allow testing the intervention in a real-world, in a country that has a significant proportion of patients with limited socioeconomic status, who do not have wide access to alternative anticoagulation therapies instead of warfarin. The perspectives of our findings include the potential to contribute for future strategies involving patients on warfarin assisted by the Brazilian Unified Health System (Sistema Único de Saúde—SUS). Besides, we believe that our findings can contribute to improve health education actions directed to low-income patients with AF treated with warfarin in other countries in the world.

## 4 Declarations

Ethics approval and consent to participate: This study has been approved by the institutional Ethics’ Committee of the Universidade Federal de Minas Gerais (UFMG), under the code CAAE 65928316.3.0000.5149. All patients will be invited to provide a signed informed consent to participate in the study. This study has been registered at Registro Brasileiro de Ensaios Clínicos (REBEC) RBR- 9cy6py and UTN: U1111-1217-0151.

## Author contributions

ACCORDING OF International Committee of Medical Journal Editors (ICMJ), the author responsibility in the study is described below:

Substantial contributions to the conception or design of the work; or the acquisition, analysis, or interpretation of study data: JMC, RER, HCB, MAPM, MSM.

Participation in drafting the work or revising it critically for important intellectual content: JMC, HCT, MAPM.

Final approval of the version to be published: JMC, MSM, HCT, RER, RPS, HCB, DDR, MAPM.

Agreement to be accountable for all aspects of the work in ensuring that questions related to the accuracy or integrity of any part of the work are appropriately investigated and resolved: JMC, MSM, HCT, RER, RPS, HCB, DDR, MAPM.

**Conceptualization:** Josiane Moreira da Costa, Milena Soriano Marcolino, Heloisa Carvalho Torres, Daniel Dias Ribeiro, Maria Auxiliadora Parreiras Martins.

**Formal analysis:** Renan Pedra de Souza.

**Funding acquisition:** Maria Auxiliadora Parreiras Martins.

**Investigation:** Josiane Moreira da Costa, Raissa Eda de Resende.

**Methodology:** Josiane Moreira da Costa, Milena Soriano Marcolino, Heloisa Carvalho Torres, Raissa Eda de Resende, Hannah Cardoso Barbosa, Daniel Dias Ribeiro, Maria Auxiliadora Parreiras Martins.

**Project administration:** Josiane Moreira da Costa, Milena Soriano Marcolino.

**Resources:** Josiane Moreira da Costa.

**Software:** Renan Pedra de Souza.

**Supervision:** Milena Soriano Marcolino, Heloisa Carvalho Torres, Daniel Dias Ribeiro, Maria Auxiliadora Parreiras Martins.

**Validation:** Milena Soriano Marcolino, Heloisa Carvalho Torres.

**Visualization:** Milena Soriano Marcolino, Heloisa Carvalho Torres, Raissa Eda de Resende, Renan Pedra de Souza, Maria Auxiliadora Parreiras Martins.

**Writing – original draft:** Josiane Moreira da Costa.

**Writing – review & editing:** Milena Soriano Marcolino, Heloisa Carvalho Torres, Raissa Eda de Resende, Renan Pedra de Souza, Hannah Cardoso Barbosa, Daniel Dias Ribeiro, Maria Auxiliadora Parreiras Martins.

Maria Auxiliadora Parreiras Martins orcid: 0000-0002-5211-411X.
